# Risk for Ocular Hypertension With Intravitreal Dexamethasone Implants in Black and White Patients With Diabetic Macular Edema

**DOI:** 10.1177/24741264241309685

**Published:** 2025-01-08

**Authors:** Ella H. Leung, Amer F. Alsoudi, Henry Chang Skrehot, Daniel Burkhead, Bradley Adcock, India Behl, David Chin Yee

**Affiliations:** 1 Georgia Retina, Atlanta, GA, USA; 2 Cullen Eye Institute, Baylor College of Medicine, Houston, TX, USA; 3 Campbell University School of Osteopathic Medicine, Lillington, NC, USA; 4 Princeton University, Princeton, NJ, USA

**Keywords:** intravitreal dexamethasone implant, ocular hypertension, race, diabetic macular edema

## Abstract

**Purpose:** To compare the effects of intravitreal (IVT) 0.7 mg dexamethasone implants on the intraocular pressure (IOP) in Black patients and White patients with diabetic macular edema (DME). **Methods:** A retrospective cohort study was performed of Black patients and White patients with DME who received dexamethasone implants with 12 or more months of follow-up. **Results:** The study included 145 eyes (69 Black; 76 White) with a mean (±SD) of 3.6 ± 3.9 dexamethasone implants and 58** ±** 31 months of follow-up. Black patients had higher baseline rates of glaucoma (23% vs 8%; *P* = .010) but similar rates of ocular hypertension after receiving IVT dexamethasone (20% vs 16%; *P* = .52). By the last follow-up visit, the mean central subfield thickness had decreased from 387** ±** 129 µm to 314** ±** 104 µm (*P* < .001). **Conclusions:** IVT dexamethasone implants decreased macular thicknesses in patients with DME; however, there was no difference in the rate of ocular hypertension after IVT dexamethasone between Black patients and White patients.

## Introduction

Intraocular steroids are effective in treating cystoid macular edema (CME) secondary to a variety of diseases, including diabetes, retinal vascular occlusions, and Irvine-Gass syndrome^1–5^; however, steroids can be associated with elevated intraocular pressure (IOP). Although the exact pathophysiology is unclear, steroids can result in direct cytotoxicity to the cells of the trabecular meshwork, triamcinolone crystals can cause obstruction of this meshwork, glycosaminoglycans can be deposited, and glucocorticoid receptors can be activated in the trabecular meshwork.^6–10^

The risk for steroid-induced glaucoma in the general population is estimated to be 8% to 36%, a risk that increases to 46% to 92% in eyes with primary open-angle glaucoma (POAG).^11^ Previous studies have found a higher prevalence of POAG in patients of African ancestry^12,13^; however, Black patients are underrepresented in clinical trials of glaucoma and other ocular diseases in the United States.^14^ In the phase 3 MEAD trials of the 0.7 mg intravitreal (IVT) dexamethasone implant (Ozurdex, Allergan) for diabetic macular edema (DME), only 4.6% of the patients were Black.^15^ Only 4% of the patients enrolled in the Ozurdex Geneva study for vein occlusions were Black.^4^ In contrast, Black people make up 14% of the US population.^16^ Compared with the previous decade, between 2011 and 2020 there were fewer Black participants in diabetic retinopathy (DR) trials, the reasons for which are unclear.^17^

Previous studies found some variable treatment responses between different races.^18–20^ Black patients with DME had fewer visual improvements after IVT bevacizumab than White and Hispanic patients, even after controlling for baseline vision and hemoglobin A_1c_.^21^ Black race was associated with an increased risk for cataract after CME and prolonged anterior uveitis after combined phacoemulsification with endoscopic cyclophotocoagulation.^22^ Black patients with uveitic glaucoma had higher treatment failures with steroids and trabeculectomies.^23^ A systematic review by Sharma et al^24^ found that preexisting glaucoma and Black race were risk factors for ocular hypertension after keratoplasty, which was related to a steroid response. IVT dexamethasone implants were associated with higher risks for steroid-induced IOP elevations in South Asian and Latino patients than in White patients; however, the study did not include Black patients.

The purpose of the current study was to compare the effects of IVT dexamethasone implants on IOP in Black patients and White patients with DME.

## Methods

A retrospective chart review was performed of all patients who received an initial IVT 0.7 mg dexamethasone implant for the treatment of DME at a large retina-only practice between January 2013 and January 2023. Exclusion criteria were no documentation of racial demographics; other etiologies for ME; a history of other IVT steroid injections, including the 0.19 mg fluocinolone acetonide IVT implant (Iluvien, Alimera Sciences) or 0.18 mg fluocinolone acetonide IVT implant (Yutiq, Alimera Sciences); less than 1 year of follow-up; and age younger than 18 years. The Sterling Institutional Review Board waived approval for the retrospective study. The study and data collection were in compliance with the provisions of the US Health Insurance Portability and Accountability Act of 1996, adhered to the tenets of Declaration of Helsinki, and complied with all country, federal, state, and local laws.

Patient demographics, including age, race and ethnicity, medical history, ocular and surgical history, ocular and systemic medications, IOP, cup-to-disc ratios, previous ocular procedures (including lasers, surgeries, and injections), treatment effects and complications, best-corrected visual acuity (BCVA), and central subfield thickness (CST), were analyzed with Prism software (version 9, GraphPad Software). Patients were grouped into the Black cohort or White cohort based on self-identification and the US Office of Management and Budget’s revisions to the Standards for the Classification of Federal Data on Race and Ethnicity. Patients who declined to report, identified as having multiracial ancestry, identified as Asians, identified as a Pacific Islander, or were of Spanish origin were excluded. The appropriate parametric or nonparametric tests were performed, and statistical significance was set at *P* < .05. The Snellen BCVA was converted to logMAR notation, with counting fingers VA being equal to 1.88 logMAR (Snellen equivalent, 20/1500), hand motions equal to 2.30 logMAR (Snellen equivalent, 20/4000),^25^ light perception equal to 2.70 logMAR, and no light perception or enucleation/evisceration equal to 3.0 logMAR. All mean values are ± SD.

## Results

A total of 145 eyes in 118 patients met the inclusion and exclusion criteria, with 69 eyes in the Black cohort and 76 in the White cohort. Table 1 shows the patients’ demographics. The Black cohort had more women and a greater proportion with hypertension. The severity of DR was similar between the 2 groups, with approximately 14% having mild nonproliferative DR (NPDR), 39% moderate NPDR, 9% severe NPDR, 39% proliferative diabetic retinopathy (PDR), and 4% tractional retinal detachments (RDs). Of the 12% of patients who had a previous vitrectomy, approximately 50% had PDR.

**Table 1.** Patient Demographics and Medical History at Baseline.^a^

Demographic	All Patients	Black Patients	White Patients	*P* Value
	(N = 145)	(n = 69)	(n = 76)	
Right eye, n (%)	167 (48)	65 (48)	102 (48)	> .99
Female, n (%)	203 (58)	87 (64)	116 (54)	.059
Mean age (y)	69.1 ± 12	68.7 ± 12	69.4 ± 11	.90
Phakic, n (%)	120 (34)	47 (35)	73 (34)	.91
Medical history				
Most recent HbA_1c_ (%), mean	7.3 ± 1.5	7.1 ± 1.3	7.5 ± 1.7	.20
Hypertension, n (%)	121 (83)	64 (93)	57 (75)	< .0001
Hyperlipidemia, n (%)	158 (45)	30 (43)	36 (47)	.64
Coronary artery disease, n (%)	21 (14)	7 (10)	17 (22)	.076
Preexisting glaucoma, n (%)				
Any glaucoma	22 (15)	16 (23)	6 (8)	.010
Primary open-angle glaucoma	4 (3)	2 (3)	2 (3)	.92
Neovascular glaucoma	1 (1)	1 (1)	0	.48
Chronic angle-closure glaucoma	4 (3)	2 (3)	2 (3)	> .99
Unspecified glaucoma	11 (8)	11 (16)	0	.0002
Steroid responder	25 (7)	0	2 (3)	.25
Previous glaucoma procedures, n (%)				
Trabeculectomy	1 (0.7)	1 (1)	0	.48
Glaucoma drainage implant	1 (0.7)	1 (1)	0	.48
Argon laser trabeculoplasty/selective argon trabeculoplasty	2 (1)	0	2 (3)	.50
Stage of DR, n (%)				
Mild NDPR	20 (14)	9 (13)	11 (14)	> .99
Moderate NPDR	56 (39)	22 (32)	34 (45)	.13
Severe NPDR	13 (9)	8 (12)	5 (7)	.39
PDR	56 (39)	30 (43)	26 (34)	.23
Tractional RD	8 (6)	4 (6)	4 (5)	> .99
Macular edema and treatments, n (%)				
Previous topical steroids	19 (13)	10 (16)	9 (12)	.81
Previous anti-VEGF	116 (80)	56 (81)	60 (79)	.84
Previous steroid injection	9 (6)	6 (7)	3 (84)	.31
Previous topical NSAIDs	7 (5)	5 (7)	2 (3)	.26
Previous PRP	19 (13)	6 (9)	13 (17)	.14
Previous vitrectomy	17 (12)	8 (12)	9 (12)	> .99
Mean duration of macular edema before dexamethasone implant (mo)	25 ± 25	27 ± 23	24 ± 26	.45
Mean number of dexamethasone implants	3.6 ± 3.9	4.2 ± 4.6	2.9 ± 3.0	.056

Abbreviations: anti-VEGF, antivascular endothelial growth factor DR, diabetic retinopathy; HbA_1c_, glycosylated A_1c_; NDPR, nonproliferative diabetic retinopathy; NSAIDs, nonsteroidal anti-inflammatory drugs; PDR, proliferative diabetic retinopathy; PRP, panretinal photocoagulation; RD, retinal detachment.

a All mean values are ± SD.

Table 2 shows the initial and final examination findings. Black patients had slightly higher baseline cup-to-disc ratios than White patients, were more likely to have preexisting glaucoma (23% vs 8%; *P* = .010, Fisher exact test), and used more glaucoma drops at the initial visit (0.54 ± 1.0 vs 0.10 ± 0.4; *P* = .0012, Mann-Whitney test). The number of previous glaucoma surgeries was similar between the 2 cohorts.

**Table 2.** Comparison of Initial and Final Examination Findings.^a^

Finding	All Patients	Black Patients	White Patients	*P* Value
	(N = 145)	(n = 69)	(n = 76)	
Mean cup-to-disc ratio in affected eye				
Initial, at 1st dexamethasone implant	0.33 ± 0.16	0.35 ± 0.13	0.29 ± 0.15	.042
Last	0.34 ± 0.16	0.38 ± 0.15	0.31 ± 0.16	.022
Change from initial to last visit	0.025 ± 0.08	0.029 ± 0.1 *P* = .014	0.022 ± 0.07 *P* = .0005	.53
Intraocular pressure (mm Hg)				
Mean baseline	15 ± 4	16 ± 4	15 ± 4	.19
Mean 4-6 weeks after 1st dexamethasone implant	17 ± 5	17 ± 5	17 ± 5	.71
Mean 3 months later	17 ± 4	17 ± 4	17 ± 5	.55
Mean 6 months later	16 ± 4	16 ± 4	16 ± 5	.83
Mean at last visit	15 ± 4	16 ± 4	15 ± 4	.25
Mean highest if ≥21 mm Hg	26 ± 5	25 ± 4	26 ± 5	.41
Mean increase ≥10 mm Hg from baseline, n (%)	26 (18)	14 (20)	12 (16)	.52
Mean increase ≥10 mm Hg in steroid responders, n (%)	3/8 (38)	2/3 (66)	1/5 (20)	.46
Mean increase ≥10 mm Hg in glaucoma patients, n (%)	4 (18)	4/16 (25)	0/6	.54
Mean increase ≥10 mm Hg after mean number of dexamethasone implants	2.5 ± 4	3.3 ± 5	1.6 ± 1	.29
Mean months to IOP elevation after last dexamethasone implant	4.7 ± 7	4.9 ± 8	3.2 ± 2	.47
Mean follow-up				
Overall months of follow-up from initial diagnosis to last clinical visit	58 ± 31	59 ± 30	58 ± 32	.95
Months of follow-up after initial dexamethasone implant	34 ± 17	32 ± 17	35 ± 18	.25
Glaucoma treatment				
Mean glaucoma medications (n)				
Drops before dexamethasone implant	0.30 ± 0.72	0.54 ± 1.0	0.10 ± 0.4	.0012
Drops at last follow-up	0.60 ± 1	0.69 ± 1	0.53 ± 1	.32
Difference in number of drops between initial and last visit	0.30 ± 0.8	0.15 ± 0.71 *P* = .14	0.43 ± 0.79 *P* < .001	.30
After dexamethasone implant, n (%)				
Trabeculectomy	0	0	0	> .99
Glaucoma drainage implant	2 (1)	2 (3)	0	.22
Mean central subfield thickness (µm)				
Before dexamethasone implant	387 ± 129	400 ± 155	375 ± 100	.29
At last visit	314 ± 104	323 ± 131	306 ± 70	.35
Change between initial and last visit	−73 ± 122, *P* < .001	−83 ± 142, *P* < .001	−69 ± 103, *P* < .001	.78
Mean BCVA (logMAR, ≈Snellen)				
Initial	0.37 ± 0.35 ≈20/47	0.36 ± 0.36 ≈20/46	0.38 ± 0.34 ≈20/48	.78
Last (logMAR, ≈Snellen)	0.42 ± 0.51 ≈20/53	0.46 ± 0.57 ≈20/58	0.38 ± 0.45 ≈20/48	.75
Change between initial and last visits	−0.002 ± 0.46, *P* = .75	−0.07 ± 0.54, *P* = .88	0.014 ± 0.39, *P* = .89	.28

Abbreviation: BCVA, best-corrected visual acuity.

a All mean values are ± SD.

Twenty-six eyes (18%) developed IOP elevations of 10 mm Hg or more above baseline during the study; the proportion was similar between Black patients and White patients (*P* = .52, Fisher test), regardless of whether the patient had a history of steroid response or underlying glaucoma. The Black cohort and White cohort had a similar number of IVT dexamethasone implants before experiencing IOP elevations (mean, 3.3 ± 5 vs 1.6 ± 1; *P* = .29, Mann-Whitney test), similar times to IOP elevations after the last dexamethasone implant (mean, 4.9 ± 8 vs 3.2 ± 2 months; *P* = .47), and similar maximum IOPs above baseline (mean, 26 ± 5 mm Hg vs 25 ± 4 mm Hg; *P* = .52). The cup-to-disc ratio increased slightly in both cohorts over the course of the study; however, the change was similar in the 2 cohorts (Table 2).

Black patients used more glaucoma drops at baseline than White patients (0.54 ± 1.0 vs 0.10 ± 0.4; *P* = .0012); however, the 2 cohorts had a similar number of drops by the last follow-up (*P* = .32) and similar initial and final IOPs (*P* = .69). Two Black patients (1%) had incisional glaucoma surgery after IVT dexamethasone implant placement; however, the difference between the 2 cohorts was not statistically significant (*P* = .22) (Table 2).

Both cohorts had a significant and similar improvement in the CST over the course of the study (387 ± 129 µm initially to 314 ± 104 µm at the last visit; *P* < .001, Mann-Whitney test); however, there was no race-based difference. Overall, there was no statistically significant difference in BCVA between the initial visit and final visit (0.37 ± 0.35 logMAR vs 0.42 ± 0.51 logMAR; *P* = .64), regardless of lens status (*P* = .98) (Table 2). The rates of adverse events, including RDs (3% Black cohort, 1% White cohort; *P* = .61), were similar between the 2 cohorts.

## Conclusions

IVT dexamethasone implants are effective in decreasing ME. They suppress inflammatory mediators and reduce angiogenesis and vascular permeability. Patients may develop elevated IOP, primarily from increased outflow resistance.^26^

The current study found that although Black patients had higher baseline and final cup-to-disc ratios and slightly higher baseline IOPs than White patients, there was no statistically significant difference in the percentage who developed ocular hypertension after dexamethasone implant placement. Approximately 18% of our patients had IOP elevations, with 1% requiring incisional glaucoma surgery. In comparison, the MEAD 3-year follow-up study found that 28% of patients developed IOP elevations of 10 mm Hg or more above the baseline, with 1.2% requiring glaucoma surgery or laser treatment.^27^ Similarly, the SAFODEX study found that 33% of patients developed IOP elevations, with 1.2% requiring filtering surgery.^28^ The CHROME study reported that 21% to 24% of patients developed IOP elevations of 10 mm Hg or higher, with 9% to 29% requiring IOP-lowering therapy.^29^

Similar to findings in previous studies, there were baseline differences in systemic and ocular comorbidities in our study, with the Black cohort being more likely to have hypertension and glaucoma.^13,30–33^ Although Black patients used more glaucoma drops initially, the 2 cohorts used a similar number of glaucoma drops by the last follow-up visit, with similar final IOPs and similar cup-to-disc ratios.

Some studies have found that patients with POAG may be more likely to have IOP elevations with steroid use, with 20% to 40% of patients having a history of steroid response.^34^ The phase 3 clinical trials of dexamethasone implants excluded patients with known histories of steroid response or glaucoma.^27^ In the current study, 15% of patients had preexisting glaucoma and 7% had a known history of ocular hypertension after steroid use; fortunately, these patients were not more likely to develop a steroid-induced IOP elevation.

The CST improved significantly in both cohorts. The mean CST improvement of −73 ± 122 µm was less than the −127 µm change reported in the MEAD trial, which may reflect that the patients in the current study had more severe and chronic DR, had persistent DME that was previously treated with more antivascular endothelial growth factor injections, may have had epiretinal membranes, and may have reached a treatment plateau.

Previous studies have noted visual improvement after IVT dexamethasone implant placement^5,15,29^; however, the current study did not. The discordance between CST improvement and a lack of visual improvement was also noted in the post hoc analysis of DRCR Protocol T, in which Black patients tended to have lower visual gains despite greater reductions in CST.^35^ The incongruity likely reflects the underlying effects of chronic ME, macular ischemia, chronic inflammation, and possible concurrent glaucomatous optic neuropathy.^21^

The limitations of the current study include its retrospective nature and lack of ancillary glaucoma testing. Additional diagnostics, such as corneal pachymetry, optical coherence tomography (OCT) of the retinal nerve fiber layers, and OCT angiography of the peripapillary vascular density and visual fields, were not performed; thus, early glaucoma changes may have been undiagnosed.^36^ The majority of IOPs were obtained with automated handheld tonometers (TonoPen, Medtronic), which may have underestimated the IOP at higher values compared with Goldmann applanation tonometer values^37,38^; however, elevated IOPs were typically confirmed with applanation tonometry.

In addition, patients were grouped according to their self-identified race; however, many patients were excluded because their race was not specified or they were from multiracial backgrounds. People of Asian or Spanish origin were excluded because of the relatively smaller samples. Race is a social, not a biological, construct, and racial associations may be related to socioeconomic health inequities.^39^ Although there was no statistically significant difference in the mean number of IVT dexamethasone implants between cohorts, there was a trend toward Black patients receiving more steroids, which may reflect a sampling bias.

In conclusion, IVT dexamethasone implants were effective at decreasing the CST; however, 18% of patients developed IOP elevations. Fortunately, most cases of ocular hypertension could be managed medically, and only 1% of patients required incisional surgery. Although in the current study, Black patients were more likely than White patients to have preexisting glaucoma, there did not appear to be a statistically significant difference in the risk for steroid-induced ocular hypertension. Larger prospective studies with ancillary glaucoma testing in more diverse patient populations would be needed to detect more subtle changes.

## References

[1] Diabetic Retinopathy Clinical Research Network. A randomized trial comparing intravitreal triamcinolone acetonide and focal/grid photocoagulation for diabetic macular edema. Ophthalmology. 2008;115(9):1447-1449, 1449.e1-10. doi:10.1016/j.ophtha.2008.06.015 18662829 PMC2748264

[bibr2-24741264241309685] Scott IU Ip MS VanVeldhuisen PC , et al. A randomized trial comparing the efficacy and safety of intravitreal triamcinolone with standard care to treat vision loss associated with macular edema secondary to branch retinal vein occlusion. Arch Ophthalmol. 2009;127(9):1115-1128. doi:10.1001/archophthalmol.2009.233 19752420 PMC2806600

[bibr3-24741264241309685] Ip MS Scott IU VanVeldhuisen PC , et al. A randomized trial comparing the efficacy and safety of intravitreal triamcinolone with observation to treat vision loss associated with macular edema secondary to central retinal vein occlusion: the Standard Care vs Corticosteroid for Retinal Vein Occlusion (SCORE) study report 5. Arch Ophthalmol Chic Ill 1960. 2009;127(9):1101-1114. doi:10.1001/archophthalmol.2009.234 PMC2872173 19752419

[bibr4-24741264241309685] Haller JA Bandello F Belfort R , et al. Randomized, sham-controlled trial of dexamethasone intravitreal implant in patients with macular edema due to retinal vein occlusion. Ophthalmology. 2010;117(6):1134-1146.e3. doi:10.1016/j.ophtha.2010.03.032 20417567

[5] Haller JA Bandello F Belfort R , et al. Dexamethasone intravitreal implant in patients with macular edema related to branch or central retinal vein occlusion twelve-month study results. Ophthalmology. 2011;118(12):2453-2460. doi:10.1016/j.ophtha.2011.05.014 21764136

[6] Roll P Benedikt O. Electronmicroscopic studies of the trabecular meshwork in corticosteroid glaucoma. Klin Monatsbl Augenheilkd. 1979;174(3):421-428. German 480814

[bibr7-24741264241309685] Sharma A Patil AJ Gupta N , et al. Effects of triamcinolone acetonide on human trabecular meshwork cells in vitro. Indian J Ophthalmol. 2014;62(4):429-436. doi:10.4103/0301-4738.121143 24817746 PMC4064217

[bibr8-24741264241309685] Zhang X Clark AF Yorio T. FK506-binding protein 51 regulates nuclear transport of the glucocorticoid receptor β and glucocorticoid responsiveness. Invest Ophthalmol Vis Sci. 2008;49(3):1037-1047. Accessed July 15, 2023. https://iovs.arvojournals.org/article.aspx?articleid=2184744 18326728 10.1167/iovs.07-1279 PMC2442563

[bibr9-24741264241309685] Steely HT Browder SL Julian MB Miggans ST Wilson KL Clark AF. The effects of dexamethasone on fibronectin expression in cultured human trabecular meshwork cells. Invest Ophthalmol Vis Sci. 1992;33(7):2242-2250. Accessed July 15, 2023. https://pubmed.ncbi.nlm.nih.gov/1607235/1607235

[10] Singh IP Ahmad SI Yeh D , et al. Early rapid rise in intraocular pressure after intravitreal triamcinolone acetonide injection. Am J Ophthalmol. 2004;138(2):286-287. doi:10.1016/j.ajo.2004.03.001 15289140

[11] Tripathi RC Parapuram SK Tripathi BJ Zhong Y Chalam KV. Corticosteroids and glaucoma risk. Drugs Aging. 1999;15(6):439-450. doi:10.2165/00002512-199915060-00004 10641955

[12] Rudnicka AR Mt-Isa S Owen CG Cook DG Ashby D. Variations in primary open-angle glaucoma prevalence by age, gender, and race: a Bayesian meta-analysis. Invest Ophthalmol Vis Sci. 2006;47(10):4254-4261. doi:10.1167/iovs.06-0299 17003413

[13] Tielsch JM Sommer A Katz J Royall RM Quigley HA Javitt J. Racial variations in the prevalence of primary open-angle glaucoma. The Baltimore Eye Survey. JAMA. 1991;266(3):369-374. 2056646

[14] Allison K Patel DG Greene L. Racial and ethnic disparities in primary open-angle glaucoma clinical trials: a systematic review and meta-analysis. JAMA Netw Open. 2021;4(5):e218348. doi:10.1001/jamanetworkopen.2021.8348 PMC8132140 34003274

[15] Boyer DS Yoon YH Belfort R , et al. Three-year, randomized, sham-controlled trial of dexamethasone intravitreal implant in patients with diabetic macular edema. Ophthalmology. 2014;121(10):1904-1914. doi:10.1016/j.ophtha.2014.04.024 24907062

[16] U.S. Census Bureau QuickFacts: United States. QuickFacts. Accessed July 15, 2023. https://www.census.gov/quickfacts/fact/table/US/PST045222

[17] Berkowitz ST Groth SL Gangaputra S Patel S. Racial/ethnic disparities in ophthalmology clinical trials resulting in US Food and Drug Administration drug approvals from 2000 to 2020. JAMA Ophthalmol. 2021;139(6):629-637. doi:10.1001/jamaophthalmol.2021.0857 33885724 PMC8063130

[18] Varma R Bressler NM Doan QV , et al. Prevalence of and risk factors for diabetic macular edema in the United States. JAMA Ophthalmol. 2014;132(11):1334-1340. doi:10.1001/jamaophthalmol.2014.2854 25125075 PMC4576994

[bibr19-24741264241309685] Wong TY Klein R Islam FMA , et al. Diabetic retinopathy in a multi-ethnic cohort in the United States. Am J Ophthalmol. 2006;141(3):446-455. doi:10.1016/j.ajo.2005.08.063 16490489 PMC2246042

[20] Bajwa A Osmanzada D Osmanzada S , et al. Epidemiology of uveitis in the mid-Atlantic United States. Clin Ophthalmol Auckl NZ. 2015;9:889-901. doi:10.2147/OPTH.S80972 PMC4445955 26056428

[21] Osathanugrah P Sanjiv N Siegel NH Ness S Chen X Subramanian ML. The impact of race on short-term treatment response to bevacizumab in diabetic macular edema. Am J Ophthalmol. 2021;222:310-317. doi:10.1016/j.ajo.2020.09.042 33045219

[22] Edmiston AM SooHoo JR Seibold LK Kahook MY Palestine AG Pantcheva MB. Postoperative inflammation after endoscopic cyclophotocoagulation: racial distribution and effect on outcomes. J Glaucoma. 2018;27(3):266-268. doi:10.1097/IJG.0000000000000884 29356715

[23] Gregory AC Zhang MM Rapoport Y Ling JD Kuchtey RW. Racial influences of uveitic glaucoma: consolidation of current knowledge of diagnosis and treatment. Semin Ophthalmol. 2016;31(4):400-404. doi:10.3109/08820538.2016.1154169 27101105

[24] Sharma A Kuppermann BD Bandello F , et al. Intraocular pressure (IOP) after intravitreal dexamethasone implant (Ozurdex) amongst different geographic populations—GEODEX-IOP study. Eye. 2020;34(6):1063-1068. doi:10.1038/s41433-019-0616-7 31570814 PMC7253432

[25] Schulze-Bonsel K Feltgen N Burau H Hansen L Bach M. Visual acuities “hand motion” and “counting fingers” can be quantified with the Freiburg visual acuity test. Invest Ophthalmol Vis Sci. 2006;47(3):1236-1240. doi:10.1167/iovs.05-0981 16505064

[26] Sarao V Veritti D Boscia F Lanzetta P. Intravitreal steroids for the treatment of retinal diseases. ScientificWorldJournal. 2014;2014:989501. doi:10.1155/2014/989501 24526927 PMC3910383

[27] Maturi RK Pollack A Uy HS , et al. Intraocular pressure in patients with diabetic macular edema treated with dexamethasone intravitreal implant in the 3-year mead study. Retina. 2016;36(6):1143-1152. doi:10.1097/IAE.0000000000001004 26871523

[28] Malclès A Dot C Voirin N , et al. Safety of intravitreal dexamethasone implant (OZURDEX): the SAFODEX study. Incidence and risk factors of ocular hypertension. Retina. 2017;37(7):1352-1359. Accessed July 15, 2023. https://pubmed.ncbi.nlm.nih.gov/27768641/27768641 10.1097/IAE.0000000000001369

[29] Lam WC Albiani DA Yoganathan P , et al. Real-world assessment of intravitreal dexamethasone implant (0.7 mg) in patients with macular edema: the CHROME study. Clin Ophthalmol Auckl NZ. 2015;9:1255-1268. doi:10.2147/OPTH.S80500 PMC4506028 26203215

[30] Nannini D Torres M Chen YDI , et al. African ancestry is associated with higher intraocular pressure in Latinos. Ophthalmology. 2016;123(1):102-108. doi:10.1016/j.ophtha.2015.08.042 26477841 PMC4695255

[bibr31-24741264241309685] Khachatryan N Medeiros FA Sharpsten L , et al. The African Descent and Glaucoma Evaluation Study (ADAGES): predictors of visual field damage in glaucoma suspects. Am J Ophthalmol. 2015;159(4):777-787. doi:10.1016/j.ajo.2015.01.011 25597839 PMC4361282

[bibr32-24741264241309685] Skaat A De Moraes CG Bowd C , et al. African Descent and Glaucoma Evaluation Study (ADAGES): racial differences in optic disc hemorrhage and beta-zone parapapillary atrophy. Ophthalmology. 2016;123(7):1476-1483. doi:10.1016/j.ophtha.2016.03.025 27117781 PMC4921256

[33] Sample PA Girkin CA Zangwill LM , et al. The African Descent and Glaucoma Evaluation Study (ADAGES): design and baseline data. Arch Ophthalmol Chic Ill 1960. 2009;127(9):1136-1145. doi:10.1001/archophthalmol.2009.187 PMC2761830 19752422

[34] Roberti G Oddone F Agnifili L , et al. Steroid-induced glaucoma: epidemiology, pathophysiology, and clinical management. Surv Ophthalmol. 2020;65(4):458-472. doi:10.1016/j.survophthal.2020.01.002 32057761

[35] Bressler SB Odia I Maguire MG , et al. Factors associated with visual acuity and central subfield thickness changes when treating diabetic macular edema with anti–vascular endothelial growth factor therapy. JAMA Ophthalmol. 2019;137(4):382-389. doi:10.1001/jamaophthalmol.2018.6786 30676635 PMC6459102

[36] WuDunn D Takusagawa HL Sit AJ , et al. OCT angiography for the diagnosis of glaucoma: a report by the American Academy of Ophthalmology. Ophthalmology. 2021;128(8):1222-1235. doi:10.1016/j.ophtha.2020.12.027 33632585

[37] Bao B Diaconita V Schulz DC Hutnik C. Tono-pen versus Goldmann applanation tonometry: a comparison of 898 eyes. Ophthalmol Glaucoma. 2019;2(6):435-439. doi:10.1016/j.ogla.2019.07.004 32672578

[38] Kao SF Lichter PR Bergstrom TJ Rowe S Musch DC. Clinical comparison of the Oculab Tono-Pen to the Goldmann applanation tonometer. Ophthalmology. 1987;94(12):1541-1544. doi:10.1016/s0161-6420(87)33249-x 3431824

[39] Flanagin A Frey T Christiansen SL , AMA manual of style committee. Updated guidance on the reporting of race and ethnicity in medical and science journals. JAMA. 2021;326(7):621-627. doi:10.1001/jama.2021.13304 34402850

